# Eliminating Uncorrected Refractive Error by 2030: A Sequential Framework for Evidence‐Based Action in LMICs

**DOI:** 10.1002/puh2.70241

**Published:** 2026-04-15

**Authors:** Indra Prasad Sharma, Kovin S. Naidoo, Khathutshelo Percy Mashige, Nor Tshering Lepcha

**Affiliations:** ^1^ Jigme Dorji Wangchuck National Referral Hospital Ministry of Health Thimphu Bhutan; ^2^ Discipline of Optometry, School of Health Sciences University of KwaZulu‐Natal Durban South Africa; ^3^ African Vision Research Institute University of KwaZulu‐Natal Durban South Africa; ^4^ Department of Optometry University of New South Wales Sydney Australia

**Keywords:** cost‐effectiveness, evidence‐based approach, framework, low‐ and middle‐income countries (LMICs), refractive error

## Abstract

Uncorrected refractive error (URE) is a leading global cause of vision impairment (VI) and remains a critical, yet under‐addressed challenge, disproportionately affecting low‐ and middle‐income countries (LMICs). In response to the substantial unmet need for refractive care, the 74th World Health Assembly (WHA) established the first global target of increasing effective refractive error coverage (eREC) by 40% by 2030. However, recent estimates show global adult eREC at 65.8% in 2023, with South Asia lagging at lower rates, indicating that no LMIC is on track. To achieve this ambitious target within the next 5 years, it is imperative to develop and implement evidence‐based, contextually adaptable, and sustainable strategies responsive to the needs of individual countries and communities. Persistent barriers, including limited resources, fragmented service delivery, and insufficient policy guidance, impede progress, particularly in LMICs. Drawing from contexts such as Bhutan, where refractive errors account for 96.1% of school‐aged VI, but effective spectacle coverage is only 11.5%, this article introduces a five‐phase, evidence‐informed framework: (1) need estimation via surveys, (2) situational analysis using tools such as WHO Refractive Error Situational Analysis Tool (RESAT), (3) model formulation, (4) evaluation and scaling, and (5) demand generation. This supports systemic planning, implementation, and scaling of sustainable refractive error services.

## 1 VIEWPOINT

### 1.1 The Burden of Uncorrected Refractive Error (URE)

URE is a leading cause of vision impairment (VI) globally and represents a critical, yet under‐addressed, component of the global eye health agenda. Enhancing access to refractive services is essential for achieving the United Nations Sustainable Development Goals (SDGs), including reducing avoidable blindness, promoting gender equity, advancing education, and boosting economic productivity [1, 2]. Given global trends in population growth, aging demographics, and lifestyle changes such as increased near work and screen time, the demand for refractive care will increase substantially in the coming decades [3, 4]. The 74th World Health Assembly (WHA) (2021) endorsed a global target to increase effective refractive error coverage (eREC) by 40% by 2030 and the WHO launched the 2030 SPECS initiative in 2024, reflecting the urgent need to address this issue.

Despite the availability of cost‐effective solutions, only 36% of individuals in need of spectacles have access to them [5, 6]. This access gap contributes to a growing economic burden in LMICs; however, scaling up URE interventions is projected to offer significant economic returns [7]. Notwithstanding the cost‐effectiveness of existing solutions, many individuals in LMICs continue to endure the preventable consequences of URE [8].

### 1.2 Systemic Barriers in LMICs

In LMICs, the provision and uptake of refractive services are hindered by multifactorial barriers. These include under‐resourced health systems, inadequate infrastructure, high cost of diagnostic equipment, fragmented spectacle supply chains, and an insufficiently trained eye health workforce. Sociocultural factors like low awareness and stigma associated with spectacle wear further impede service utilization [9]. Refractive services are often not integrated into national public health frameworks, resulting in URE being a low priority in health agendas, with corresponding funding gaps, accessibility, and policy attention. It is further exacerbated by marked rural–urban disparities and economic constraints. Additionally, the scarcity of baseline data and context‐specific evidence complicates strategic planning and monitoring of progress. The COVID‐19 pandemic worsened these strains, intensifying the challenges associated with URE service delivery, and delaying progress toward eREC targets. Emerging issues, such as the increasing use of digital screens in LMICs, add complexity.

### 1.3 Current Approaches and Their Limitations

Governments, civil society organizations, and philanthropies are actively engaged in developing and testing efficient and high‐quality models of refractive care delivery [10]. Various approaches currently considered include public sector‐led services, NGO‐driven programs, social entrepreneurship models, private sector initiatives, and public–private sector partnerships, each offering distinct advantages and limitations. While promising, these models often lack universal adaptability and desired scale, often failing to address equity or sustainability [10].

As countries progress toward attaining the eREC target, the burden of URE is expected to increasingly concentrate within marginalized and underserved populations. This epidemiological shift poses both challenges and potential opportunities. Addressing URE in these populations necessitates sustained, coordinated efforts underpinned by collaborative, innovative, and multisectorial strategies. Key components of such strategies include securing political commitment, promoting international collaboration, mobilizing financial investment, strengthening health system infrastructure, expanding the training and deployment of eye care professionals, and harnessing digital technologies, like tele‐refraction, to improve access to and the efficiency of refractive care delivery. Long‐term sustainability will depend on the integration of innovative, inclusive business models with cost‐effective technological solutions [11].

Implementing and scaling URE initiatives in LMICs is challenging, primarily due to resource constraints and contextual variability. Uniform or “one‐size‐fits‐all” strategies are ineffective, necessitating countries to recalibrate their interventions based on demographics, infrastructure, and public–private contributions. The complexity of URE demands a structured, phased framework to design, operationalize, and scale context‐specific, sustainable interventions that address each LMIC's unique health system and socioeconomic realities.

### 1.4 Proposed Sequential Framework

**FIGURE 1:**
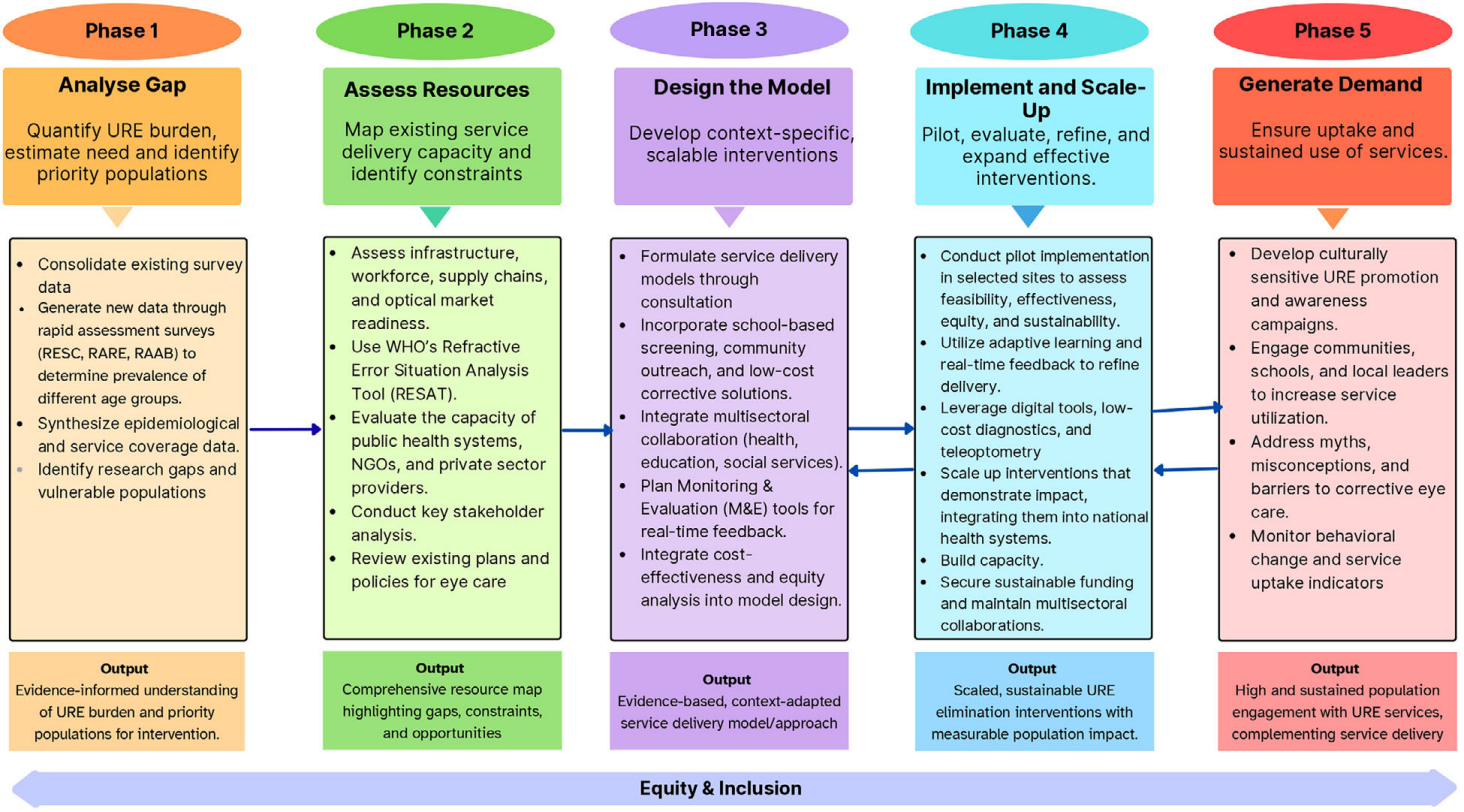
A proposed sequential framework for developing an evidence‐based approach to eliminate uncorrected refractive error.

To support LMICs in this endeavor, we propose a structured, sequential framework designed to guide the formulation of an evidence‐informed model and the development of a contextually appropriate implementation strategy tailored to the target population. This framework comprises five interrelated phases, each informed by the outcomes of the preceding stage: (1) estimation of population‐level need, (2) assessment of existing resources and service delivery capacity, (3) formulation of a fit‐for‐purpose model or approach, (4) evaluation of the model's effectiveness, followed by its implementation, and (5) demand generation (Figure 1).

Phase 1 necessitates a comprehensive assessment of the magnitude and nature of the problem, including an estimation of the target population's needs. This involves the generation and synthesis of robust, population‐level prevalence data, including eREC, by employing rapid assessment methodologies [12]. These findings enable identification of vulnerable populations, help close research gaps, and form the evidentiary basis for the subsequent phases. In Phase 2, a situational analysis could be conducted using the WHO Refractive Error Situational Analysis Tool (RESAT) [13] to systematically map infrastructure, workforce, and capacity for service delivery. This will help identify existing service gaps, barriers to access, and potential opportunities within the eye health system. Insights from these first two phases, along with a review of best practices, will guide the subsequent phases.

In Phase 3, a context‐specific and scalable model should be developed, integrating public awareness, systematic screening, and provision of accessible, high‐quality refractive services. The model must define core components of URE intervention; target populations, strategies, and expected outcomes, while ensuring affordability, availability, and adherence to corrective eyewear. Strengthening infrastructure, workforce capacity, and intersectoral collaboration is essential, alongside enabling policy support. Robust monitoring and evaluation mechanisms should be embedded from the outset to guide performance and impact.

Phase 4 involves piloting the model, evaluating and implementing the model, leveraging adaptive learning, and providing feedback to refine delivery and monitor outcomes. Pilot implementation and iterative refinement should be embedded in the model's development phase to identify and address contextual barriers prior to broader scale‐up. It should then be integrated within the health system and aligned with broader policy frameworks to facilitate scale‐up and sustainability. In Phase 5, the focus should shift to generating demand to ensure that services are matched by uptake. Culturally sensitive awareness campaigns, community and school engagement, and behavior‐change monitoring are essential.

Collectively, this five‐phase framework provides an actionable, data‐driven, and equity‐sensitive pathway—shaped by real‐world constraints and guided by policy imperatives—to eliminate URE by 2030 in LMICs. Implementation requires an equity focus, addressing gender and rural disparities through inclusive models. Effective implementation of the model may require capacity building among healthcare providers and the establishment of multisectoral collaboration to enhance delivery, coordination, and impact of services.

### 1.5 Framework Implementation Considerations for Bhutan

In Bhutan, a landlocked Himalayan LMIC, the framework illustrates practicality. Bhutan's strong commitment to universal health coverage and its well‐organized primary health system provide a solid foundation for eliminating URE. Bhutan is a signatory to the WHA eREC endorsement and launched the national 2030 SPECS initiative in July 2025.

In Phase 1, Bhutan can build upon existing RAAB 2018 and Bhutan School Sight Survey 2019 and conduct a fresh RARE survey to generate up‐to‐date estimates of prevalence and eREC. Additionally, national health information systems can be strengthened to bridge the data gap. Stratifying these data by region, age, and socioeconomic group will help identify priority populations, particularly school‐aged children and underserved rural communities. In Phase 2, Bhutan could employ WHO's RESAT to map service readiness, human resources, and supply chains at the national level. It could highlight gaps in policies, human resources, and optical dispensing facilities. This could inform a SWOT analysis of refractive services and resources in Bhutan.

Designing the model would require a context‐adapted, hybrid approach. School‐based vision screening can be expanded nationwide with trained teachers or school health coordinators as first‐level screeners, supported by optometrists and technicians at district and regional hospitals. Community‐based outreach, particularly in remote areas, could be integrated into mobile health teams. Ensuring affordability and accessibility of corrective eyewear will be critical, potentially through subsidized spectacle provision within the public system, complemented by regulated private sector engagement.

Moving to Phase 4, Bhutan could begin by piloting the model in selected districts, using adaptive learning to refine the model. Successful models can subsequently be scaled nationally through their integration into the Ministry of Health's essential health services package. Concurrently, investment in workforce training would ensure sustainability. This could include incorporating vision screening and basic refraction modules in the training curriculum of health assistants and other allied health professionals at Khesar Gyalpo University of Medical Sciences of Bhutan. Partnerships with NGOs and international agencies could support financing and technical inputs during the scale‐up phase.

Finally, to generate demand for refractive services, Bhutan would require culturally sensitive health promotion campaigns that align with its Gross National Happiness philosophy. These efforts could address misconceptions about spectacle wear, mobilize schools and community leaders, and leverage mass and social media platforms to normalize refractive error correction. Monitoring service uptake and public attitudes would ensure that demand generation remains responsive and impactful.

### 1.6 Path to Achieving 2030 Targets

While achieving the eREC target by 2030 is ambitious, it remains achievable. LMICs must shift from fragmented strategies to contextually responsive, data‐driven models. Piloting this framework in Bhutan could inform LMIC models, and urge global collaboration.

## Author Contributions

**Indra Prasad Sharma**: conceptualization, methodology, investigation, writing – review and editing, and writing – original draft. **Kovin S. Naidoo**: conceptualization, investigation, supervision, writing – review, and final critical review. **Khathutshelo Percy Mashige**: methodology, investigation, supervision, and writing final critical review. **Nor Tshering Lepcha**: methodology, investigation, supervision, writing – original draft, and final critical review.

## Funding

The authors have nothing to report.

## Conflicts of Interest

The authors declare no conflicts of interest.

## Data Availability

Data sharing not applicable to this article as no datasets were generated or analyzed during the current study.
